# Gay/Lesbian sexual orientation increases risk for cigarette smoking and heavy drinking among members of a large Northern California health plan

**DOI:** 10.1186/1471-2458-6-241

**Published:** 2006-10-03

**Authors:** Elisabeth P Gruskin, Nancy Gordon

**Affiliations:** 1 Kaiser Permanente Division of Research 2000 Broadway, 3^rd ^Floor Oakland, CA 94612, USA

## Abstract

**Background and significance:**

Tobacco and alcohol use and related morbidity and mortality are critical public health problems. Results of several, but not all, studies suggest that lesbians and gay men are at elevated risk for smoking tobacco and alcohol misuse.

**Methods:**

Data from random sample general health surveys of adult members of a large Northern California Health Plan conducted in 1999 and 2002 were analyzed using gender-based multivariate logistic regression models to assess whether lesbians (n = 210) and gay men (n = 331) aged 20–65 were more likely than similarly aged heterosexual women (n = 12,188) and men (n = 9342) to be smokers and heavy drinkers.

**Results:**

After adjusting for age, race/ethnicity, education, and survey year, lesbians were significantly more likely than heterosexual women to be heavy drinkers (OR 2.14, 95% CI 1.08, 4.23) and current smokers (OR 1.60, 95% CI 1.02, 2.51). Among men, gays were significantly more likely than heterosexuals to be current smokers (OR 2.40, 95% CI 1.75, 3.30), with borderline significant increased risk for heavy drinking (OR 1.54, 95% CI 0.96, 2.45).

**Conclusion:**

Lesbians and gay men may be at increased risk for morbidity and mortality due to higher levels of cigarette and alcohol use. More population-based research is needed to understand the nature of substance use in these communities so that appropriate interventions can be developed.

## Background

Tobacco-related diseases are the most preventable health problems in the United States. Although tobacco use is declining, it is responsible for nearly 20% of the deaths in the U.S., and kills approximately 50% of continuous smokers. Smoking-related diseases include many types of cancer, chronic obstructive pulmonary disease, coronary heart disease, stroke, peripheral vascular disease and peptic ulcer disease [1]. Lung cancer is the leading cause of cancer-related death among women and men in the United States [2].

Similarly, alcohol misuse may contribute to morbidity and mortality resulting in acid-related disorder, arthritis, asthma, cirrhoses of the liver, diseases of the pancreas, hepatitis C, chronic obstructive pulmonary disease, diabetes, hypertension, injuries and overdoses, depression, anxiety, and major psychosis [3,4]. If, as many studies indicate, lesbians and gay men have higher rates of smoking and alcohol misuse than heterosexuals, it is likely that these segments of the population will also have higher rates of cancer, respiratory disease and cardiovascular morbidity than similarly aged heterosexuals.

Same-gender sexual orientation and sexual behavior have been identified as risk factors for smoking among women [5-15] in population-based studies [10-13,16], convenience samples with internal comparison groups [14,17] and convenience samples with outside comparison groups [5-9,15]. Only two published studies, Gruskin et al. 2001 and Dibble et al 2004 [16,18], found risk of current smoking was not significantly different for older lesbians compared to heterosexuals. Similarly, multivariate analyses of population-based studies [19-21] and studies relying on convenience samples [9,22] have found that gay men and men who have sex with men are more likely to be current smokers than heterosexual men.

Evidence about the relationship of sexual orientation with risk for alcohol problems is more mixed. Previous studies have found lesbians to be more likely to have alcohol binges [10,23] than heterosexual women and/or women who have sex with men only, to be dependent on alcohol [24-28], and to experience negative consequences related to alcohol [25,26,29-33]. In addition, they were more likely to seek help for alcohol-related problems and/or to be in recovery [5,26,29,30]. However, several studies have not found differences by sexual orientation and/or sexual behavior [17,18,34,35], and others suggest that if there is an association, it may be restricted to certain age groups [16,31].

Studies on alcohol use post-1995^1 ^are also mixed for gay men. Some studies have found no difference in alcohol use between gays and heterosexuals [21,24,26,27,36,37]. Other studies found gay men more likely to report frequent heavy drinking and less likely to be abstinent from alcohol than heterosexual men [9,22,31-33,38].

Although there have been recent improvements in research about sexual orientation and alcohol and tobacco use, significant limitations remain. Some of the population-based studies included fewer than 100 lesbians and/or bisexual women or gays and/or bisexual men [12,27,29,34,36,39], limiting the statistical power. While studies specifically targeting lesbians or gays have had larger samples, they have lacked comparison data obtained using the same methodology and instruments, making comparisons problematic. Also, many studies that used sexual behavior as a proxy for sexual orientation focused on recent sexual behavior, hence, misclassifying or eliminating people who were not sexually active in the given time interval.

The current study has several advantages when compared with previous research. We used data from two random sample surveys of the adult membership of the Kaiser Permanente Medical Care Program in Northern California. A participant will not be included in both databases, even if they are picked twice. Only one person in each household was chosen to participate in the survey. Combined they yielded a sample of 12,188 heterosexual women, 210 lesbians, 9342 heterosexual men and 331 gay men aged 20–64, resulting in 1.6% lesbians and 3.4% gay men. These numbers are similar to those of the current research. The most recent methodological strong study using a probability sample found that there were 1.4% lesbians and 3.2% gay men [13]. In a study conducted by Drabble et al,, only .9% reported being lesbian and 1.7% reported being gay [26]. Diamant et al, found 1.1% of her sample to be lesbian[12]. The members of this prepaid health plan described in this study are sociodemographically representative of the non-Medicaid insured adult population in Northern California. Large numbers of participants enabled sufficient statistical power for meaningful comparisons between groups and to conduct multivariate analyses. Finally, the survey asked respondents to identify their sexual orientation rather than sexual behavior, resulting in a sample that includes people who identify as lesbian or gay but who may not have recently been sexually active.

This study is particularly important because it does not use a typical convenience sample and because it clarifies some of the results for gay men which are mixed.

## Methods

In April-July of 1999 and 2002, general health surveys were mailed to independent stratified random samples of 40,000 female and male adult members of the Kaiser Permanente Medical Care Program in Northern California. Survey eligibility was limited to current health plan members aged 20 years or older, who were not known to have a non-English language preference and who were not cognitively impaired. Surveys were sent up to three times to enhance the response rate. The Kaiser Foundation Research Institute Institutional Review Board approved this study, The response rates for women and men aged 20–64 (the age cut-offs for the current study) were 45% and 35%, respectively, in both survey years, yielding a final sample of 210 lesbians, 331 gay men, 12,188 heterosexual women, and 9342 men.

The survey questionnaire covered sociodemographic characteristics, health and functional status, health-related behaviors including tobacco and alcohol use, and selected aspects of health care use.

The sexual orientation question in the 1999 survey was worded: "What is your sexual orientation?" The response categories were: (1) "heterosexual ("straight")"; (2) "homosexual (gay, lesbian)"; and (3) "other." In 2002, the question was worded slightly differently: "Are you gay, lesbian or bisexual?" The response categories were: (1) "no"; (2) "yes, gay/lesbian"; and (3) "yes, bisexual." To enable pooling of respondents for both survey years, we restricted our final sample to women who indicated that they were either heterosexual or lesbian and men who indicated that they were gay or heterosexual.

The tobacco questions were worded: "Have you ever regularly smoked cigarettes (that is, smoked daily for at least a year)?" and "Do you smoke cigarettes now?" Based on responses to these questions, we created the outcome variables "current smoker" and "current or former smoker." We created a variable to represent heavy drinking based on responses to two questions that assessed drinking frequency and number of drinks usually consumed at a time. The frequency question was: "During the past 12 months, how often have you had a drink containing alcohol?" with response options of "almost every day," "5 to 6 times a week," "3 to 4 times a week," "1 to 2 times a week," "2 to 4 times a month," "1 time a month or less," "Never in the past 12 months." The follow-up consumption question was worded "On days when you had a drink, how many drinks did you usually have?" An average weekly alcohol consumption score was created by multiplying usual number of drinks by the midpoint of the weekly drinking frequency category, with people drinking less than once a week coded to reflect none or a fraction of a week. Heavy or problem drinking was defined based on usual number of drinks at a time (4 for women and 5 for men) or total consumption of more than twenty-one drinks a week [40].

Respondents were categorized as depressed if they indicated that they had experienced depression or extreme sadness that had lasted for at least two weeks in past 12 months or that they had taken an antidepressant during the past 12 months. They were categorized as being highly stressed if they indicated that they had felt very stressed, tense, or anxious much or most of the time during the past 12 months.

### Statistical analysis

The combined respondent sample was weighted post-stratification to match the age (by 5-year intervals), gender and geographic distribution of the adult membership at the time the 2002 sample was pulled. All analyses were done using weighted data. To assess whether sexual orientation was associated with smoking and heavy drinking, we used gender-specific multiple logistic regression models that controlled for age (continuous variable of 5-year age intervals), race-ethnicity (indicator variables for African American/Black, Latino/Hispanic, Asian, Pacific Islander, and Other, with White nonHispanics as the reference group), educational attainment (indicator variables for <12 years, some college, college graduate, with high school graduate as the reference group), and the survey year from which the responses came (2002 vs. 2001), all of which were statistically significant bivariate predictors of smoking status and heavy drinking. All analyses were performed using SAS version 9.2 Proc Surveyfreq, Proc Surveymeans, and Proc Surveylogistic procedures for data derived from complex survey designs. [41]

## Results

A comparison of the demographic characteristics of the heterosexual and gay/lesbian samples is shown in Table 1. Both the lesbians and gays had significantly higher percentages of White nonHispanics and college graduates than the heterosexuals, with no significant difference in mean age. They were also more likely to have been depressed or under treatment for depression, and the gay men were significantly more likely to be highly stressed.

### Association of sexual orientation with smoking and heavy drinking

Before adjusting for sociodemographic differences, the percentage of current smokers was not significantly different for the lesbian group (14.5%, CI: 9.2%–19.8%) and heterosexual women (11.5%, CI: 10.9%–12.1%). However, after adjusting for age, race/ethnicity, and education (see Table 2), lesbians were significantly more likely to be smokers (OR: 1.60, CI: 1.02–2.51). Lesbians were also more likely to report having ever regularly smoked than heterosexual women (43.2% vs. 31.2%, respectively, adjusted OR: 1.86, CI: 1.35–2.55). Similarly, while the unadjusted percentages of heavy drinkers did not significantly differ between lesbians (5.9%, CI: 2.3%–9.5%) and heterosexual women (3.2%, CI: 2.9%–3.6%), after adjusting for demographics, lesbians were significantly more likely to be heavy drinkers (OR: 2.14, 95% CI 1.08–4.23).

Gay men were significantly more likely to be current smokers (23.6%, CI: 18.4%–28.8%) than heterosexual men (14.7%, CI: 13.9%–15.6%), with an adjusted OR of 2.40, (CI 1.75–3.30). There was no significant difference in percentages of heavy drinkers between gay men (9.3%, CI: 5.5%–13.1%) and heterosexual men (7.1%, CI: 6.5%–7.7%), although gay men had a borderline significant increased risk after adjusting for demographics (OR: 1.54, CI 0.96–2.45).

Because smoking tends to be much less common among college-educated adults, we made a closer examination of the relationship of sexual orientation with smoking among men and women who were college graduates, adjusting for age, race/ethnicity, and survey year. Among male graduates, we found being gay was associated with a threefold increase in risk of being a smoker (OR = 3.5, CI: 2.3–5.5), a much larger difference than observed for lesser levels of education. Among women, likelihood of smoking associated with sexual orientation was consistent across all levels of education.

## Discussion

The results of our study of differences in smoking and heavy drinking between heterosexual and gay/lesbian members of a large Northern California health plan are in accord with previous research that suggests gays and lesbians are at higher risk for use of these substances than their heterosexual counterparts. The difference in smoking risk for lesbians is not as large as has been found in other studies. This is likely due in part to the fact that overall, a significantly lower percentage of adults in the San Francisco Bay area smoke compared with in other parts of the country, and in part to the fact that the lesbians in our sample were better educated than the heterosexual women. Additionally, the expansive efforts being made by Kaiser Permanente to encourage smokers to quit smoking and nonsmokers not to start may be contributing to the narrowing of differences between heterosexuals and lesbians. However, there was a larger difference in risk of smoking between gay and heterosexual men, especially among those who are college educated. While we found that gays were significantly more likely to be depressed and highly stressed than heterosexual men, these psychosocial factors did not attenuate the relationship of sexual orientation with smoking among college-educated men when added into a multiple logistic regression model controlling for age, race/ethnicity, and survey year (available from the first author).

The strength of this study is that the gays, lesbians, and heterosexuals being compared are drawn from the same large probability sample of health plan members rather than convenience samples or comparisons of samples drawn from entirely different populations. We also controlled for sociodemographic and negative affect differences between the lesbians/gays and heterosexual segments of the population which have been shown to affect likelihood of smoking and heavy drinking in the general population. There are enough variables to control for in multivariate analyses, which showed different patterns than the bivariate analyses.

However, our study still has some limitations. First, despite the fact that our study had relatively large samples of lesbians and gay men compared with previous random sample population-based surveys, the numbers of gays and lesbians were relatively small and skewed toward the higher end of the socioeconomic spectrum as compared to the heterosexual respondents., and we do not know the sociodemographic characteristics of the people who did not fill out the survey or did not fill out the question on sexual orientation. Second, only one question was used to define sexual orientation. We may have miscategorized men and women who have sex with someone of the same gender but do not self-identify as lesbian or gay, and because of wording differences in the sexual orientation question on the survey, we needed to exclude people who are bisexual or transgender. Finally, any gays and lesbians who were unwilling to disclose their sexual orientation would not have been included in the study.

## Conclusion

Gays and lesbians appear to be at higher risk than heterosexuals for smoking, and lesbians, but not gay men, for heavy drinking. This is despite the fact that in this study population, the gay and lesbian samples were better educated than the heterosexual comparisons. However, it is still unclear why this is so, especially since as a group they tend to be better educated It is important to conduct targeted studies of this segment of the population, not only to monitor whether trends in use of these substances mirror those in the general population, but also to learn whether the motivations of lesbians and gays to engage in these behaviors differ from those of heterosexuals. The results of such surveys are necessary for developing and evaluating the effectiveness of general and targeted campaigns and programs to reduce smoking and excessive drinking in these communities.

## Pre-publication history

The pre-publication history for this paper can be accessed here:



## Figures and Tables

**Table 1:** Characteristics of Heterosexual and Gay/Lesbian Samples^1^

	Women		Men	
Characteristic	Heterosexual N = 12,188 Wtd. % (Unwtd %)	Lesbian N = 210 Wtd. % (Unwtd %)	Heterosexual N = 9,342 Wtd. % (Unwtd %)	Gay N = 331 Wtd. % (Unwtd %)

Age Group*				
20–29 yr	21.1 (15.8)	12.2 (8.6)	20.6 (11.7)	12.6 (7.0)
30–39 yr	23.6 (22.3)	28.4 (25.7)	24.6 (20.2)	28.9 (24.8)
40–49 yr	25.1 (26.4)	33.9 (36.2)	25.2 (25.2)	34.9 (37.2)
50–59 yr	21.8 (23.5)	21.6 (22.9)	21.4 (26.9)	18.7 (21.8)
60–65 yr	8.4 (12.0)	4.0 (6.7)	8.2 (15.5)	4.8 (9.4)
Mean age (s.e.)	41.7 (0.1) (43.7)	41.9 (0.7) (43.6)	41.6 (0.1) (45.7)	41.9 (0.6) (44.5)
				
Race/Ethnicity*				
White/non-Hispanic	64.2 (65.9)	76.1 (78.3)	63.9 (67.6)	77.2 (79.3)
African-American/Black	7.0 (7.3)	6.0 (5.4)	5.7 (6.3)	3.0 (3.1)
Latino(a)/Hispanic	12.4 (11.7)	10.2 (8.9)	12.4 (10.8)	11.7 (9.6)
Asian/Pacific Islander	16.0 (14.8)	6.6 (6.4)	17.5 (14.8)	7.8 (7.7)
Other	0.4 (0.4)	1.1 (1.0)	0.5 (0.5)	0.3 (0.3)
				
Educational Attainment*				
Non-High School Graduate	3.2 (3.5)	2.5 (1.9)	4.5 (4.8)	1.0 (1.2)
High School Graduate	13.9 (14.4)	5.0 (4.3)	14.4 (14.4)	5.9 (5.7)
Some College	44.0 (43.9)	32.9 (32.4)	40.0 (39.9)	32.9 (31.7)
4-Yr College Graduate	38.9 (38.2)	59.6 (61.4)	41.1 (41.0)	60.1 (61.3)
				
Experienced depression for ≥ 2 weeks or took anti-depressant during past 12 mos.	16.2 (16.5)	22.5* (22.9)	7.4 (7.9)	23.9* (23.3)
Felt very stressed, tense, or anxious much of the time	25.1 (23.9)	28.5 (27.0)	16.8 (15.8)	23.7* (23.2)

* Difference between weighted percents significant at p < .05 by Rao-Scott chi-square test.

**Table 2:** Association of Sexual Orientation with Current Smoking and Heavy Drinking After Adjusting for Demographic Characteristics^1^

Characteristic	Women		Men	
	Current Smoker Adj. OR (95% CI)	Heavy Drinker Adj. OR (95% CI)	Current Smoker Adj. OR (95% CI)	Heavy Drinker Adj. OR (95% CI)

Sexual Orientation				
Heterosexual	(ref gp)	(ref gp)	(ref gp)	(ref gp)
Lesbian/Gay	1.60 (1.02–2.51)	2.14 (1.08–4.23)	2.40 (1.75–3.30)	1.54 (0.96–2.45)
Race/Ethnicity				
White nonHispanic	(ref gp)	(ref gp)	(ref gp)	(ref gp)
African-American/Black	1.23 (0.99–1.52)	0.55 (0.34–0.89)	0.93 (0.91–1.02)	0.82 (0.51–1.31)
Latino(a)	0.48 (0.38–0.60)	0.66 (0.48–0.89)	0.64 (0.50–0.81)	1.70 (1.30–2.22)
Asian	0.45 (0.35–0.56)	0.41 (0.26–0.63)	0.98 (0.80–1.20)	0.54 (0.38–0.77)
Pacific Islander	0.81 (0.32–2.07)	0.30 (0.04–2.26)	3.26 (1.61–6.60)	2.28 (0.98–5.33)
Other	1.11 (0.81–1.52)	0.92 (0.49–1.71)	1.33 (0.96–1.84)	0.94 (0.57–1.57)
Educational Attainment				
< 12 years	1.22 (0.89–1.52)	1.50 (0.86–2.64)	1.46 (1.09–1.96)	0.86 (0.57–1.32)
High School Graduate	(ref gp)	(ref gp)	(ref gp)	(ref gp)
Some College	0.73 (0.62–0.86)	0.77 (0.56–1.04)	0.73 (0.61–0.87)	0.67 (0.52–0.87)
4-Yr College Graduate	0.25 (0.21–0.31)	0.41 (0.29–0.57)	0.26 (0.21–0.32)	0.38 (0.28–0.50)

^1 ^Adjusted odds ratios and confidence intervals from multiple logistic regression models using weighted survey data and also adjusting for age (continuous variable of 5-yr intervals) and survey year
